# Factors Affecting Purchase Intention of Hanfu: Considering Product Identification, Cultural Motivation, and Perceived Authenticity

**DOI:** 10.3390/bs13080689

**Published:** 2023-08-19

**Authors:** Xianyue Li, Yongge Niu, Jiuping Xu

**Affiliations:** 1 Business School, Sichuan University, Chengdu 610064, China; xianyueliscu@163.com; 2 Department of Marketing and E-Commerce, Business School of Sichuan University, Chengdu 610064, China; niuyongge@scu.edu.cn

**Keywords:** traditional clothing, Hanfu, cultural motivation, identification with product, authenticity, purchase intention

## Abstract

With the trend of national cultural confidence and the growing appreciation for aesthetic diversity, traditional apparel from different countries or regions has become a driving force in the clothing industry. Hanfu, an emerging trend that industrializes traditional culture, has garnered increasing attention from consumers. Thus, with the objective of exploring the psychological antecedents of Hanfu consumers’ purchase intentions from the perspectives of product identification, cultural motivation, and consumers’ perceived authenticity, the present study was empirically conducted with a sample of 823 respondents. Partial least squares-structural equation modeling (PLS-SEM) was employed to examine the proposed research model. The results demonstrate that consumers’ identification with Hanfu and cultural motivation positively influence object-based and existential authenticity, as well as purchase intention. Furthermore, the mediating effect of perceived object-based authenticity is confirmed, indicating its significance in shaping consumers’ purchase intentions, while the mediating effect of existential authenticity is found to be insignificant. Research findings could contribute to the understanding of the psychological mechanisms driving consumers’ purchase intentions towards traditional clothing and highlight the importance of consumers’ perceived object-based authenticity in the market of traditional cultural clothing.

## 1. Introduction

The rise of China-Chic, driven by the development of domestic product consumption and the revitalization of Chinese traditional culture, has sparked interest across various industries in leveraging national culture and branding to attract consumers. In the apparel industry, one notable phenomenon is the increasing popularity of Hanfu, the traditional clothing of the Chinese people which serves as a prominent representative of traditional Chinese culture [1]. Hanfu has evolved from being associated with feudal conservatism to becoming a representative part of contemporary fashion culture [2,3]. It has transitioned from being a niche industry appealing to a small group of individuals interested in cultural revitalization to an emerging fashion trend in the clothing industry. The market size of Hanfu is estimated to be 12.54 billion CNY (approximately 1.8 billion USD) in 2022, with an annual growth of 23.4%, and is expected to continue expanding, given its potential to capture an even larger share [4]. As an essential cultural product, Hanfu has garnered increasing attention from both academic and industry practitioners [5,6].

Prior studies have demonstrated that intrinsic motivation and congruity interact to strengthen consumers’ perceptions of brand authenticity [7]. Additionally, qualitative research by O’Neal explored how traditional dress serves as an expression and definition of the “self” and helps individuals connect with their heritage, as observed in African American professional women’s motivation for embracing ethnic dress [8]. Building on the notion that consumers are prone to purchase products which are congruent with their self-image [9,10,11], previous research has shown that consumers’ identification with products positively influences their purchasing intentions, particularly in niche industries such as green consumption and traditional food [12,13]. In modern society, Hanfu represents not only apparel but also consumers’ expression of themselves and desire for cultural and historical connotations. Therefore, consumers’ identification with Hanfu and cultural motivation, which encompasses their needs and desires related to Hanfu, become critical constructs for marketing strategies, including Hanfu positioning and market segmentation [14]. Therefore, consumers’ identification with Hanfu and their cultural motivation towards Hanfu could be taken as psychological determinants which drive the form of purchase intention. As such, one of the objectives of the present study is to empirically explore and understand the psychological mechanism of consumers’ purchase intentions from the angles of their identification with Hanfu and cultural motivation.

Authenticity plays a pivotal role in the consumers’ product attitude formation process [15]. Previous studies have examined authenticity in various domains, including art [16,17], cuisine [18,19], traditional food [13], tourism [20], and even sports [21]. Moreover, Ko et al. have verified that consumers’ perceptions of authenticity have sizable effects on consumers’ generation of preferences or positive images towards products containing traditional culture [22]. Kolar and Zabkar developed an authenticity model based on consumer perspective in the context of cultural heritage marketing and empirically demonstrated that cultural motivation is one of the antecedents influencing consumers’ perceived authenticity [23]. Compared to the significance of authenticity in culture-related consumption, especially in cultural tourism such as heritage [24] and theme parks [25], the application of the consumer-based authenticity model to unravel the consumption of traditional clothing containing cultural connotation, such as Hanfu, has been limited thus far. Hence, another objective of the present study is to apply the consumer-based authenticity model to understand the forming mechanism of consumers’ purchase intentions towards Hanfu.

Collectively, this research develops and empirically tests a theoretical model which explores the correlations among consumers’ identification with Hanfu, cultural motivation, perceived object-based and existential authenticity, and their purchase intentions towards Hanfu. Moreover, the mediating effect of consumers’ perceived authenticity between consumers’ identification with Hanfu/cultural motivation and purchase intentions will be examined. Consequently, this study addresses the following research questions: (1) Do consumers’ identification with Hanfu and cultural motivation positively influence their purchase intentions? (2) What are the effects of consumer-perceived object-based and existential authenticity in the relationship between consumers’ identification with Hanfu/cultural motivation and purchase intentions? (3) Does consumers’ object-based authenticity positively affect their perceived existential authenticity?

## 2. Literature Review and Hypotheses Development

### 2.1. Identification with Hanfu

Products are seen as reflections of consumers’ personalities, and the symbolic meaning of a certain product often embodies more significance compared to its functional aspects. Previous studies have shown that consumers prefer products that align with their self-image or the image they want others to perceive [9,10]. As a result, consumers continuously shape their identities through their consumption choices of specific products, services, or brands. Clothing, in particular, holds the power to not only serve as an object but also as an expression of identity, values, and emotions [26]. Consumers construct their sense of self through clothing and fashion [27], and the color of clothing has been found to influence individuals’ sexual self-cognition and attitudes [28]. Furthermore, Van der Laan and Velthuis suggested that clothing functions as a vehicle to demonstrate individuals’ identity [29]. Therefore, the identification with the clothing one wears can directly or indirectly impact the psychological mechanisms of decision-making.

Authenticity, as defined by Fleeson and Wilt and Sheldon et al., is a state in which one’s behaviors align with their core traits or abilities, reflecting their “true self” [30,31,32]. Studies on niche products, such as traditional food, have shown that consumers who identify more strongly with the product experience higher levels of subjective authenticity [13]. Additionally, the relationship between players’ identification with their avatars in gaming and their perceived authenticity has been demonstrated, suggesting that the emotional and cognitive connection between players and their avatars influences their perceptions of authenticity [33,34]. Furthermore, for consumers’ perceived authenticity in the context of tourism, He et al. have synthesized three dimensions of tourists’ perceived authenticity, and found that the perceived consistency, which is similar to the concept of identification, was one of the most significant facets [35].

In this context, Hanfu, as a cultural and historical apparel, goes beyond its functional purpose and becomes intertwined with consumers’ emotional and psychological needs. It serves as a means for consumers to express their personalities, self-cognition, and cultural interactions through external symbols and status items [6,27]. Based on the above discussion, when consumers form identification with Hanfu, their purchase intentions would be increased because of the congruity between themselves and the particular product. Furthermore, their perceptions of authenticity could be activated. Therefore, the following hypotheses regarding consumers’ identification with Hanfu, object-based and existential authenticity, and their purchase intentions can be developed:

**H1a:**  *Consumers’ identification with Hanfu positively affects their perceived object-based authenticity.*

**H1b:**  *Consumers’ identification with Hanfu positively affects their perceived existential authenticity.*

**H1c:**  *Consumers’ identification with Hanfu positively affects their purchase intentions.*

### 2.2. Cultural Motivation

Consumers’ motivation for purchasing certain products refers to their needs, desires, and wants for the product [14], and it is a vital concept for understanding consumers’ behavior [36]. Ajzen proposed that intention, which could capture motivational factors affecting consumer behavior, emphasizes individuals’ efforts to engage in a behavior [37]. In the context of cultural apparel, cultural motivation refers to consumers’ motives and desires to purchase or wear Hanfu, driven by their interest in the cultural aspects associated with it. Cultural motivation plays a significant role in consumers’ consumption of Hanfu and can be seen as the foundation for market segmentation and positioning strategies [14].

Cultural motivation is associated with intrinsic elements of motivation, influencing their perceptions [38,39]. Therefore, it can be inferred that consumers’ cultural motivations could positively impact on their perceived object-based and existential authenticity. Additionally, based on the conceptual framework established by Kolar and Zabkar, cultural motivation toward cultural heritage positively affects consumers’ perceived existential authenticity, object-based authenticity, and the consequent loyalty of tourists [23]. Yildiz et al. also highlighted that tourists’ cultural travel motivation is the most significant predictor of their satisfaction [40]. Based on these insights, the following hypotheses are proposed:

**H2a:**  *Consumers’ cultural motivations toward Hanfu positively affect their perceived object-based authenticity.*

**H2b:**  *Consumers’ cultural motivations toward Hanfu positively affect their perceived existential authenticity.*

**H2c:**  *Consumers’ cultural motivations toward Hanfu positively affect their purchase intentions.*

### 2.3. Object-Based Authenticity and Existential Authenticity

Consumers’ judgment or perceptions of authenticity is a central concern when evaluating cultural products [41,42]. According to the consumer-oriented approach, authenticity is an evaluative judgment based on consumers’ perceptions of a specific object [43,44]. Consumer-perceived authenticity is generally differentiated into existential authenticity and objective authenticity [45]. Objective authenticity refers to the genuineness or accurate representation of physical or observable aspects like the peculiarities, cultural elements, and design of Hanfu [46,47].

Existential authenticity, on the other hand, is associated with consumers’ subjective feelings and experiences that enhance their sense of connection and self-presentation [48]. Hanfu, as a type of traditional Chinese clothing, not only represents a reproduction of genuine or accurate objects but also deeply embodies historical and cultural meanings which could be perceived by direct involvement [6]. Therefore, consumers’ perceived authenticity toward Hanfu can be understood from the angles of object-based and existential authenticity.

Previous studies by Kolar and Zabkar [23], Atzeni et al. [49], Yi et al. [50], and Yildiz et al. [40] have demonstrated the positive influence of object-based authenticity on existential authenticity concerning cultural tourism. Moreover, the significant correlations between consumers’ or tourists’ authenticity and various aspects of their decision-making have been empirically examined and verified. For example, museum visitors’ perceived authenticity was found to affect their post-visitation behavior [48], and consumers’ purchase intentions toward traditional restaurants were influenced by their perceived authenticity related to local authentication and chain ownership [51]. In accordance with the above discussion, research hypotheses are proposed as follows:

**H3a:**  *Consumers’ perceived object-based authenticity toward Hanfu positively affects their existential authenticity.*

**H3b:**  *Consumers’ perceived object-based authenticity toward Hanfu positively affects their purchase intentions.*

**H4:**  *Consumers’ perceived existential authenticity toward Hanfu positively affects their purchase intentions.*

### 2.4. Mediating Effect

Previous studies have investigated and verified the direct influences of consumers’ cultural motivations or identification with products in different contexts on behavioral intentions, such as satisfaction, loyalty, or purchase/travel intention [23,33,52,53]. Additionally, the mediating effect of consumers’ perceived authenticity has been explored in the literature. For example, Biraglia et al. found that changes in the physical or atmospheric attributes of a tourist attraction could affect perceived authenticity, which in turn influenced tourists’ intention to visit [54]. Yildiz et al. demonstrated that both object-based and existential authenticity mediate the relationship between tourists’ cultural travel motivation and satisfaction [40]. Shoenberger and Kim have verified that consumers’ perceived authenticity towards an influencer would mediate the relationship between their perceptions of homophily and subsequent purchase intentions [55]. However, in the context of traditional and cultural apparel consumption, such as Hanfu, the mediating role of consumers’ perceived object-based and existential authenticity in the relationship between their motivation or identification with Hanfu and their purchase intentions has received limited attention. Considering the nature of findings in comparative literature, which suggests the presence of variables mediating the relationship between consumer motivation/identification with products and purchase intentions, it is essential to explore and reveal the mediating role in the context of current research [23,33,51]. Therefore, we develop the following hypotheses:

**H5a:**  *Consumers’ perceived object-based authenticity mediates the relationship between consumers’ identification with Hanfu and purchase intentions.*

**H5b:**  *Consumers’ perceived existential authenticity mediates the relationship between consumers’ identification with Hanfu and purchase intentions.*

**H6a:**  *Consumers’ perceived object-based authenticity mediates the relationship between consumers’ cultural motivations toward Hanfu and purchase intentions.*

**H6b:**  *Consumers’ perceived existential authenticity mediates the relationship between consumers’ cultural motivations toward Hanfu and purchase intentions.*

**Figure 1:**
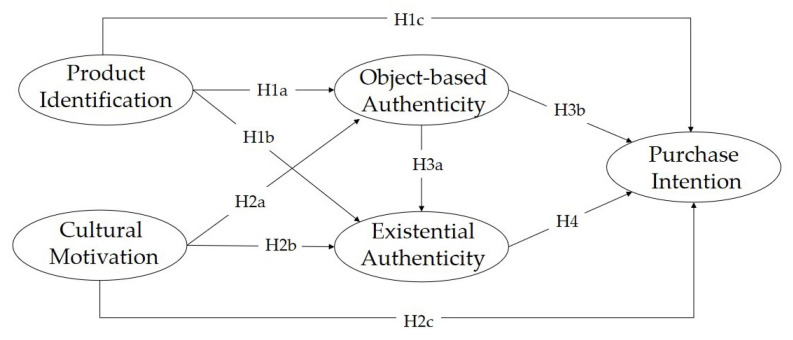
Proposed research framework.

In summary, the objective of this study is to empirically investigate the relationship among consumers’ identification with Hanfu, cultural motivation, perceived object-based and existential authenticity, and purchase intentions. Furthermore, the mediating roles of two types of perceived authenticity in the paths between identification/motivation and purchase intention, respectively, will be examined. The developed research model is illustrated in Figure 1.

## 3. Methodology

### 3.1. Sample and Procedure

A quantitative methodology was performed to empirically examine the hypothesized relationships among research variables, that is, consumers’ identification with Hanfu, cultural motivation, object-based authenticity, existential authenticity, and purchase intentions [2,56,57]. To ensure the questionnaire’s cultural relevance and validity, a double translation method suggested by Kivela et al. was employed [58]. The questionnaire, initially designed in English, was first translated into Chinese by a professional translator who was bilingual in English and Chinese. Then, another professional translator, who was also bilingual, translated the Chinese version back into English. The authors reviewed both translations, considering the Chinese cultural context, conceptual meaning, and construct validity, to finalize the Chinese version of the questionnaire.

Previous to the official data collection procedure, the Chinese version of the questionnaire was reviewed by two Hanfu consumers with experience in purchasing Hanfu and two researchers familiar with the consumer-based authenticity model and consumer behavior. Based on their feedback, minor adjustments were made to ensure clarity and ease of understanding for respondents. The final agreement was made among the authors and four participants concerning the final version of the questionnaire.

The multiple-stage sampling process was employed to collect responses from potential respondents. Firstly, a Hanfu sales company that maintained a directory of approximately 20,000 Hanfu consumers was approached to be taken as the source of potential responses to the survey. Secondly, based on the directory of the company, a random sampling method was conducted to randomly select 1500 consumers who had purchased Hanfu within the past year as the study sample. The sampling procedure was conducted with the assistance of the company’s staff. To administer the questionnaire, an online survey platform called Wenjuanxing (https://www.wjx.cn/ (accessed on 20 September 2022)) or Questionnaire Star was employed. The platform provided a unique URL link and QR code for participants to access the questionnaire. Ethical approval was obtained from the ethics committee of the university that the authors worked at before conducting the study to ensure the validity of this study in terms of the ethical dimensions. In the meantime, participants were assured of confidentiality and sole academic use of data.

The questionnaire was accessible from 10 December 2022 to 13 March 2023, until no new responses were generated. A total of 1082 samples were initially collected. After the screening, 259 questionnaires were deleted due to short completion time (less than 250 s) (191) or straight-line answers (68). As a result, 823 rational responses were obtained, yielding a valid response rate of 76.1%. The sample size of 823 was considered appropriate for performing data analysis using PLS-SEM, as recommended by Hair et al. (2011) for research frameworks with five or fewer latent constructs, with each latent construct containing more than three measurement items.

The socio-demographic characteristics of respondents are indicated in Table 1. Among the valid 823 collected samples, 573 were female (69.6%) and 250 were male (30.4%). In terms of respondents’ age, respondents between 25–34 years old accounted for the largest group, with a total of 560 (68%) respondents in this age group, followed by the group between 18 and 24 years old with 118 respondents (14.3%). Regarding their educational background, 78.6% of respondents hold a bachelor’s degree. In terms of monthly income, 31.8% of respondents reported an income of over 15,000 CNY, followed by 20.3% with an income ranging from 8500 to 10,999 CNY and 15.6% with an income of 11,000 to 14,999 CNY. Moreover, the spatial scope of respondents was synthesized based on the IP addresses provided by Wenjuanxing and they were categorized into seven major geographical regions of China [59]. The results showed that 213 (25.6%) respondents were from East China, followed by 132 respondents (15.87%) from Northeast China and 121 (14.54%) from Central China. This geographical distribution of respondents is quite consistent with the statistics provided by iiMedia Report, which demonstrate that most consumers of Hanfu are from the first-tier or new first-tier cities such as Beijing (North China), Shanghai, and Hangzhou (East China) [4].

### 3.2. Measures

The structured survey questionnaire comprises of two major sections. The first section contains questions regarding respondents’ socio-demographic characteristics such as their age, gender, educational background, etc. The second section focuses on measuring the research constructs, including the independent variables of “product identification” and “cultural motivation”, the mediating variables of “object-based authenticity” and “existential authenticity”, and the dependent variable of “purchase intention.” A five-point Likert scale was used, varying from 1, representing “strongly disagree”, to 5, standing for “strongly agree”, to assess respondents’ judgement of each item related to the research constructs.

The operationalization of the research variables was as follows. For the construct of “identification with product”, four items were adapted from studies by Laura Sidali and Hemmerling [13] and Wu and Hsu [33], such as “Wearing Hanfu is one way to express myself” and “Hanfu fits with my style.” To measure consumers’ “cultural motivation” towards Hanfu, five items were used, adapted from research by Kolar and Zabkar [23] and Poria et al. [60]. Examples of these items include “Wearing Hanfu is my way to show my interest in history” and “I can have a good time with my friends who also like Hanfu.”

Consumers’ “perceived authenticity” was measured using two constructs: “existential authenticity” and “object-based authenticity.” Ten items were adopted from studies by Atzeni et al. [49], Kolar and Zabkar [23], and others. Examples of five items for measuring “existential authenticity” include “I felt connected with human history and civilization when I wear Hanfu” and “Wearing Hanfu enriched me as a person.” Examples of five items for measuring “object-based authenticity” include “I like the historical meaning connected to Hanfu” and “The Hanfu I wear is truly historical and cultural clothing.”

The dependent variable of “purchase intention” was evaluated with five items adapted from studies by Aucouturier et al. [61] and Bian and Forsythe [62]. Examples of these items include “I will (further) purchase Hanfu in the future” and “The probability I would consider buying Hanfu is high.” An overview of research variables and relative references was synthesized in Table 2 (specific measuring items were indicated in Table A1).

### 3.3. Data Analysis

IBM SPSS Statistics (Version 26) and SmartPLS 3.2.9 software were used for data analysis. Following the recommendation of Hair et al. [63], PLS-SEM was conducted for hypothesis testing, as it allows for predicting key drivers and maximizing the variance of dependent constructs. PLS-SEM is particularly useful for exploratory studies and extending existing theoretical frameworks through examining the measurement model and structural model. The measurement model examines the relationships between the constructs and their indicators, while the structural model reveals the relationships among the latent constructs within the research framework.

## 4. Results

### 4.1. Normality, Non-Response Bias, and Common Method Bias

Descriptive analysis using SPSS was conducted to testify the normality of research data. It was calculated that the kurtosis and skewness values of the items were within the acceptable range (lower than critical values of ±2.58 and ±1.96, respectively). However, the *p*-value of the Kolmogorov–Smirnov (K-S) test was less than 0.05, indicating that the sample data violated the assumption of normal distribution, although it is worth noting that the normal distribution assumption is not crucial for PLS-SEM analysis.

Harman’s one-factor test was conducted to ascertain the common method bias. According to Podsakoff et al. [64], if a single component explains 50% or more of the covariance among research variables, this indicates the existence of common method bias. Exploratory factor analysis (EFA) results revealed that no single component explained more than 31.87% of the variance, suggesting there is no common method bias issue.

At last, nonresponse bias was examined by comparing the 823 participants who returned valid questionnaires with the 677 participants who did not return valid questionnaires using the Chi-square test for demographic variables such as age and gender [65]. Consequently, no significant differences exist between the two groups in demographic variables, confirming the absence of nonresponse bias.

### 4.2. Overall Model Assessment

Overall model assessment was evaluated by the index of goodness of fit (GoF). The GoF index, varying from 0 to 1, represents the geometric mean of the average communality and average $R^{2}$ for all endogenous constructs [66]. A threshold value of 0.50 for communality and different effect sizes of $R^{2}$ can be utilized to determine the GoF [67,68]. According to Wetzels et al. (2009), a GoF value exceeding 0.36 refers to large effect size, and the calculated GoF index in this study is 0.497, higher than the value of a large effect size, confirming the good fit of the established research model [68].

### 4.3. Evaluation of the Measurement Model

As proposed by Hair et al., the reflective measurement model was evaluated from four major parameters, that is, internal consistency, indicator reliability, convergent validity, and discriminant validity [69].

First, the factor loadings of indicators were examined to assess reliability and it was found that all factor loadings exceed the recommended value of 0.6, indicating an acceptable reliability (see in Table A2). Second, Cronbach’s alpha (CA) and composite reliability (CR) were used to assess the internal consistency. As results, except for the identification with the product, all other constructs had values of CA and CR higher than the threshold value of 0.70. Although the CA of “identification with product” is 0.640, which is slightly lower than 0.7, it is still considered to be acceptable [69,70]. Therefore, the internal consistency and composite reliability of the proposed research model have been ensured.

Third, the convergent validity was examined by AVE values (see in Table 3). AVE values above 0.50 were considered indicative of convergent validity, except for the identification with product construct [69]. Due to the low factor loading of IP3, its AVE did not meet the cutoff value. As a result, IP3 was eliminated from the construct, improving the convergent validity of the construct.

As shown in Table 3, the square root of the AVE for each latent construct (diagonal values) in the correlation matrix exceeds the correlation coefficients of latent constructs, confirming the discriminant validity according to the Fornell and Larcker criterion [67]. Furthermore, the discriminant validity is fulfilled by HTMT ratio (the values in the brackets in Table 3) with which all values of HTMT are lower than the recommended threshold of 0.85 [71]. Finally, cross-loadings of items were examined, and it was observed that the item loadings of the constructs were higher than the loadings of their corresponding indicators, confirming the reliability and validity of the overall measurement model (see in Appendix B) [72]. Overall, the reliability and validity of the proposed research model have been fulfilled.

### 4.4. Structural Model Evaluation

The structural model was assessed using coefficients of determination (*R*^2^), predictive relevance (*Q*^2^), effect sizes (*f*^2^), and effect sizes (*q*^2^) [69]. The coefficients of determination (*R*^2^) indicate the explanatory power of the endogenous constructs. *R*^2^ values of 0.75, 0.50, and 0.25 are considered large, moderate, and weak, respectively [73]. The values of *R*^2^ and *R*^2^*_adj_* both unearth the moderate predictive accuracy or explanatory power of the endogenous constructs of the research model. The results show that identification with product and cultural motivation explained 37.5% of the variance in object-based authenticity. Identification with Hanfu, cultural motivation, and object-based authenticity explained 38.1% of the variance in existential authenticity. In addition, 56.8% of the variance of purchase intention was explained by other variables in the model. For the evaluation of collinearity of structural models, it is necessary to consider the combination of each dependent variable in the structural model and multiple predictors (independent variables) that predict that dependent variable. Multiple predictors (independent variables) that are the same dependent variable should not have variance inflation factor (VIF) values higher than 5 [74]. In this study, there are three dependent variables (object-based authenticity, existential authenticity and purchase intention) with two or more predictors. For object-based authenticity, the VIF values of the two predictors were both 1.165 and 1.165. The VIF values of the three predictors of existential authenticity were 1.190, 1.657 and 1.600. The VIF values of the four predictors of purchase intention were 1.244, 1.881, 1.667 and 1.617. These VIF values are all less than the critical value of 5, so there is no obvious collinearity problem.

The blindfolding procedure was conducted to examine the predictive relevance (*Q*^2^) of the model. As suggested by Hair et al., the *Q*^2^ are treated as small with values lower than 0.02, medium with values ranging from 0.02 to 0.15, and large with values higher than 0.35 [69]. Thus, the predictive relevancies of the model constructs are all medium (see *Q*^2^ values in Table 4).

**Figure 2:**
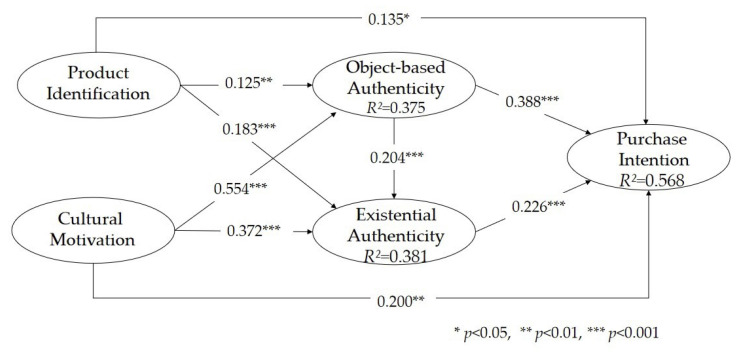
The structural model.

In order to examine the hypothesized correlations among research variables, a bootstrapping procedure with 5000 subsamples was performed, and the results can be seen in Figure 2. In addition, Table 5 represents the estimates of the structural model covering the path coefficients, effect size (*f*^2^), which is an instrumental parameter for evaluating the strength of any statistical claim, *t*-values, and level of significance (*p*-values). Based on Hair et al., *f*^2^ values of 0.02, 0.15, or 0.35 explain an exogenous construct’s small, moderate, or large effect on an endogenous construct, respectively [69]. As results, all hypothesized relationships were found to be statistically significant. Moreover, according to the values of *f*^2^, H1a, H1b, H1c, H2b, H2c, H3a, and H4 were attested to be small effects, H3b was attested to be a moderate effect, and H2a was found to be a large effect.

### 4.5. Results of the Mediation Test

The mediating roles of object-based and existential authenticity were examined using 5000 bootstrap samples. The results of mediation tests are summarized in Table 6. To evaluate the mediating role of the mediators, variance accounted for (VAF) was used. The values of VAF were calculated based on the formula proposed by Hair et al. of total direct effect divided by total indirect effects [73]. According to Nitzl et al., VAF values higher than 80% indicate full mediation, values ranging from 20% to 80% indicate partial mediation, and values lower than 20% indicate no mediation [75]. The mediation effect of the path (identification with product → object-based authenticity → purchase intention) accounted for 20.87%, indicating partial mediation. The mediation effect of the path (identification with product → existential authenticity → purchase intention) accounted for 17.83%, indicating no mediation. The mediation effect of the path (cultural motivation → object-based authenticity → purchase intention) accounted for 40.95%, indicating partial mediation. The mediation effect of the path (cultural motivation → existential authenticity → purchase intention) accounted for 16.00%, indicating no mediation.

## 5. Conclusions

In line with the research objectives, the validated research model examining consumers’ identification with Hanfu, cultural motivation, perceived authenticity, and purchase intentions provides valuable insights into the psychological mechanisms driving consumers’ purchase intentions in the niche market of Hanfu. The results obtained from a sample of 823 Hanfu consumers demonstrate significant relationships and shed light on the factors influencing consumers’ purchase intentions towards Hanfu.

The findings reveal that both consumers’ identification with Hanfu and their cultural motivation have a positive impact on their perceived object-based and existential authenticity, as well as their purchase intentions. Specifically, consumers’ cultural motivations exerts a stronger influence on object-based authenticity (*β* = 0.554, *p* < 0.001) and existential authenticity (*β* = 0.372, *p* < 0.001) compared to the effect of consumers’ identification with Hanfu on object-based authenticity (*β* = 0.125, *p* < 0.01) and existential authenticity (*β* = 0.183, *p* < 0.001). These results suggest that consumers’ cultural motivation plays a crucial role in shaping their purchase intentions in the context of culture-related products, highlighting the need to consider cultural motivation in marketing strategies. Furthermore, the positive and direct effects of consumers’ identification with Hanfu (*β* = 0.135, *p* < 0.05) and cultural motivation (*β* = 0.200, *p* < 0.01) on purchase intentions are empirically validated, supporting the significant roles of consumers’ identification with Hanfu in their consumption of Hanfu. These findings align with existing literature on the positive impact of identification with products/brands on decision-making processes [13,33,76].

Additionally, the study confirms that consumers’ perceived object-based authenticity has a positive effect on their existential authenticity (supported by H3a), consistent with prior research by Atzeni et al. [49] and Kolar and Zabkar [23]. However, other studies, such as Park et al. [77] and Laura Sidali and Hemmerling [13], have reported different findings regarding the impact of objective authenticity on existential authenticity in the contexts of heritage tourism and traditional food consumption, respectively. The results suggest that for investigations focused on traditional cultural clothing, the positive effect of object-based authenticity on existential authenticity should be emphasized, indicating that consumers’ perceptions of the historical and cultural elements embedded in Hanfu influences their authenticity experience.

The study further examines the positive effects of both object-based authenticity (*β* = 0.388, *p* < 0.001) and existential authenticity (*β* = 0.226, *p* < 0.001) on consumers’ purchase intentions (supported by H3b and H4, respectively). Comparing the path coefficients, it is evident that object-based authenticity has a stronger impact on purchase intention than existential authenticity. This finding is consistent with the mediating results, which show that consumers’ object-based authenticity mediates the relationship between their identification with Hanfu/cultural motivation and purchase intentions (supported by H5a and H6a). In contrast, the mediating effect of existential authenticity is found to be insignificant (rejected by H5b and H6b). This result aligns with the findings of Yildiz et al., highlighting the significance of object-based authenticity as a crucial antecedent variable [40]. It implies that consumers’ identification with Hanfu and cultural motivation enhance and reinforce object-based authenticity, thereby motivating their purchase intentions. In other words, consumers’ purchase intentions is driven by their identification with Hanfu and cultural motivation, leading to their perceptions of object-based authenticity in Hanfu.

## 6. Discussions

Based on the research findings, the research could provide significance for academia and contribute to the development of the Hanfu industry. Specifically, the theoretical implications and practical implications were discussed as follows.

### 6.1. Theoretical Implications

This study contributes to the existing literature in several theoretical aspects. Firstly, the empirical findings provide evidence for the positive relationship between consumers’ identification with Hanfu, cultural motivation, object-based and existential authenticity, and purchase intentions in the context of cultural apparel consumption. This research paradigm can serve as a foundation for future studies investigating the psychological antecedents of purchase intention towards cultural apparel. In contrast to previous research that focused on ethnic identity to understand consumers’ engagement with ethnic-inspired designs [78], this study establishes the empirical link between consumers’ identification with the specific product itself and their purchase intentions. This finding emphasizes the importance of considering consumers’ identification with a particular product in understanding their purchase intentions towards cultural products. This notion has also been strengthened by research in various contexts, such as food consumption, through which consumers indicate their inner self, and in the sector of tourism, where tourists’ perceived consistency is a dimension of perceived authenticity and would positively impact their travel intentions [35,79].

Secondly, regarding the relationship between consumers’ identification with Hanfu and the two types of perceived authenticity, it is observed that consumers’ identification with Hanfu has a more significant influence on their existential authenticity compared to its impact on object-based authenticity. This can be explained by the conceptualization of existential authenticity as a unique state of being true to oneself and acting authentically, rather than conforming to public roles and expectations [80]. Consistent with studies by Cook et al. [12] and Laura Sidali and Hemmerling [13], consumers tend to consume products with which they can identify to express their true selves. However, it is found that existential authenticity does not mediate the relationship between consumers’ identification with Hanfu and purchase intentions, indicating that consumers’ perceptions of existential authenticity does not significantly enhance their purchase intentions. On the other hand, the mediating role of object-based authenticity in the association between consumers’ identification with Hanfu/cultural motivation and purchase intentions is supported, suggesting that consumers’ perceptions of object-based authenticity has a positive effect on their purchase intentions. Therefore, further efforts and research are needed to refine the conceptualization of object-based authenticity to comprehensively capture its impact on consumers’ purchase intentions [40]. Moreover, different from prior studies which examined perceived authenticity as a whole to verify its mediating role on the association between consumers’ perceptions of homophily and subsequent purchase intentions [55], the present study has testified the mediating roles of perceived authenticity from two different dimensions. The discrepancy concerning the mediating effects of perceived object-based authenticity and existential authenticity could provide empirical evidence for future research to further explore the conceptualization of perceived authenticity.

Thirdly, the study confirms the positive correlations between consumers’ cultural motivations and both types of perceived authenticity, aligning with the findings of Kolar and Zabkar in the context of cultural heritage marketing [23]. This suggests the practical application of the tourist-based model of authenticity in the field of traditional clothing consumption. Moreover, it is found that consumers’ cultural motivations have a stronger impact on their perceived authenticity compared to the effect of their identification with Hanfu. This indicates that consumers are strongly driven by their cultural motivation when engaging with culture-related products, and further research is instrumental to reveal the decision-making process of consumers in the consumption of cultural products.

Lastly, the confirmed reliability and validity of the proposed research model indicate that the measurement instruments effectively capture consumers’ perceived object-based and existential authenticity in the context of cultural product consumption. This extends the application of the consumer-based authenticity model to the domain of traditional cultural apparel. Furthermore, the results highlight the central role of consumers’ perceived object-based authenticity as a link between their identification with Hanfu/cultural motivation and their purchase intentions. This underscores the importance of considering consumers’ perceptions of object-based authenticity in understanding their purchase intentions towards cultural products.

### 6.2. Practical Implications

This study has explored the psychological factors that influence Hanfu consumers’ purchase intentions based on the consumer-based authenticity model, and could provide several practical implications for Hanfu marketing, The following managerial implications can be derived from the findings.

Firstly, considering the two types of perceived authenticity, it is instrumental and essential to recognize the mediating role of object-based authenticity in the relationship between consumer identification with Hanfu/cultural motivation and purchase intentions. This suggests that the cultural and historical elements incorporated into Hanfu are clearly perceived by consumers, influencing their purchase intentions. Therefore, when designing and promoting Hanfu, it is crucial to highlight and emphasize the specific cultural and historical meanings through precise and targeted elements or designs. Rather than solely focusing on the existential connection with Hanfu, attention should be given to the cultural and historical aspects that resonate with consumers, enhancing their perceived object-based authenticity.

Secondly, the study highlights the direct influence of consumers’ cultural motivations on object-based and existential authenticity as well as purchase intentions. Consumers’ strong interest in culture acts as a significant predictor of their positive behavior towards Hanfu. In light of this, Hanfu practitioners should develop strategies to respond to and cater to consumers’ cultural and intellectual motives. Effective marketing strategies could involve emphasizing the cultural connotation of Hanfu and raising awareness prior to consumers’ consumption. For example, collaborations with cultural heritage sites to organize Hanfu festivals or initiatives to enhance consumers’ understanding of Hanfu’s cultural, spiritual, and historical significance through revival movements can be implemented.

Lastly, consumers’ identification with Hanfu influences their purchase intentions based on their perceptions of authenticity. Consumers wear Hanfu to create diverse images that reflect how they want others to perceive them. This result is very similar to the research findings verified by Morhart et al. that a brand should provide identity-relevant features and offer means of self-verification, implicitly illuminating the significance of reflecting consumers’ perceived self-image in Hanfu design [76]. Hence, stakeholders in the Hanfu industry should enhance the attractiveness of Hanfu by improving its design, incorporating cultural elements, ensuring high-quality craftsmanship, and promoting diversity. These measures can effectively enhance consumers’ purchase intentions towards Hanfu.

### 6.3. Limitations and Future Research

Though the study has been completed, it is important to acknowledge several limitations that could provide insights for future research and potential improvements. Consistent with the perspectives proposed by Kolar and Zabkar [23] and Leigh et al. [81], the use of a single measurement for the complex, multifaceted, and controversial concept of authenticity may have limitations in fully capturing its nuances. Therefore, future research should focus on the further development and promotion of authenticity conceptualization by adopting dynamic and holistic perspectives through multi-staged approaches which would contribute to a more comprehensive understanding of authenticity and its effects.

Additionally, it is worth noting that the extended consumer-based authenticity model, incorporating consumers’ identification with a specific product, was applied for the first time in the context of Chinese Hanfu to explore the internal mechanisms underlying consumers’ purchase intentions towards Hanfu consumption. Therefore, caution should be given when generalizing the research findings to other cultural products, particularly in industries different from traditional clothing, such as tea consumption in China or traditional cuisine in various countries. Moreover, diverse dimensions of perceived authenticity, such as perceived integrity and consistency [35], could be further considered to obtain a more precise understanding of the forming mechanism of purchase intention toward Hanfu. However, the findings of this study still can be a valuable reference for future research examining products with cultural or historical meanings.

At last, the impact of respondents’ spatial distribution or ethnicity was not considered in the present study, which may have an effect on consumers’ purchase intentions because people of different ethnicities or from different regions may vary in cognition towards Hanfu. Hence, more specific differentiation marketing strategies could be garnered by recognizing the ethnicity of consumers regarding the consumption of cultural products. Collectively, it would be instrumental for future research to consider the moderating effect of consumers’ ethnic characteristics on the relationship between motivational factors and their purchase intentions or their perceived authenticity towards Hanfu.

## Figures and Tables

**Table 1:** Descriptive statistics of respondents.

Variables	Categories	Frequency	Percentage%
Gender	Male	250	30.40%
	Female	573	69.60%
Age	18–24 years	118	14.30%
	25–34 years	560	68.00%
	35–44 years	113	13.70%
	45–54 years	26	3.20%
	55 years or above	6	0.70%
Educational Level	High school or below	7	0.90%
	Diploma	9	1.10%
	Bachelor	647	78.60%
	Master or above	160	19.40%
Occupation	Government officials or institution workers	57	6.90%
	Company employee	667	81.00%
	Businessman	2	0.20%
	Students	83	10.10%
	Freelancer	10	1.20%
	Others	4	0.50%
Monthly Income (CNY)	3500 or below	71	8.60%
	3500–5999	70	8.50%
	6000–8499	125	15.20%
	8500–10,999	167	20.30%
	11,000–14,999	128	15.60%
	15,000 or above	262	31.80%
Areas	North China	123	14.78%
	Northeast China	58	6.97%
	East China	213	25.60%
	Central China	121	14.54%
	South China	99	11.90%
	Northeast China	132	15.87%
	Southwest China	86	10.34%

**Table 2:** Constructs and corresponding references.

Constructs	No. of Items	References
Product Identification	4	[13,33]
Cultural Motivation	5	[23,60]
Existential Authenticity	5	[13,23,49]
Object-based Authenticity	5	[13,23]
Purchase Intention	5	[61,62]

**Table 3:** Results of reliability and validity tests.

	CA	rho_A	CR	AVE	IP	CM	OA	EA	PI
IP	0.709	0.705	0.837	0.632	**0.795**	(0.501)	(0.425)	(0.527)	(0.576)
CM	0.788	0.792	0.856	0.547	0.377	**0.739**	(0.747)	(0.721)	(0.785)
OA	0.812	0.816	0.869	0.572	0.333	0.601	**0.756**	(0.614)	(0.827)
EA	0.776	0.778	0.849	0.531	0.392	0.564	0.489	**0.729**	(0.749)
PI	0.757	0.752	0.838	0.511	0.428	0.611	0.663	0.581	**0.715**

CM = cultural motivation, EA = existential authenticity, IP = identification with product, OA = object-based authenticity, PI = purchase intention. Diagonal elements in bold are the square root of the AVE. The off-diagonal elements are the correlations among latent variables. The HTMT value is in brackets.

**Table 4:** Results of *R*^2^, adjusted *R*^2^, and *Q*^2^.

Constructs	*R* ^2^	*R* ^2^ *_adj_*	*Q* ^2^
Object-based Authenticity	0.375	0.373	0.212
Existential Authenticity	0.381	0.379	0.198
Behavioral Intention	0.568	0.566	0.273

**Table 5:** Hypothesis test results.

	Hypotheses	*f* ^2^	*t*-Value	*p*-Value	Result
H1a: IP → OA	0.125	0.021	3.170	0.002	Accepted
H1b: IP → EA	0.183	0.046	4.279	<0.001	Accepted
H1c: IP → PI	0.135	0.034	2.420	0.016	Accepted
H2a: CM → OA	0.554	0.422	16.794	<0.001	Accepted
H2b: CM → EA	0.372	0.135	8.020	<0.001	Accepted
H2c: CM → PI	0.200	0.049	2.924	0.003	Accepted
H3a: OA → EA	0.204	0.042	4.130	<0.001	Accepted
H3b: OA → PI	0.388	0.209	7.271	<0.001	Accepted
H4: EA → PI	0.226	0.073	4.841	<0.001	Accepted

CM = cultural motivation, IP = identification with product, EA = existential authenticity, OA = object-based authenticity, PI = purchase intention.

**Table 6:** Results of the mediation test.

Hypothesized Relationships	Direct Effect	Indirect Effect			VAF (%)	Result
		*β*	*t*-Value	*p*-Values		
H5a IP → OA → PI	*β* = 0.135, *t* = 2.420, *p* = 0.016	0.048	2.689	0.007	20.87	partial mediation
H6a IP → EA → PI		0.041	2.758	0.006	17.83	no mediation
H5b CM → OA → PI	*β* = 0.200, *t* = 2.924, *p* = 0.003	0.215	6.527	<0.001	40.95	partial mediation
H6b CM → EA → PI		0.084	3.797	<0.001	16.00	no mediation

IP = identification with product, CM = cultural motivation, OA = object-based authenticity, EA = existential authenticity, PI = purchase intention, VAF > 80%: full mediation, 20% ≤ VAF ≤ 80%: partial mediation, VAF < 20%: no mediation.

## Data Availability

Data is available on request due to privacy restrictions. The data presented in this study are available on request from the corresponding author.

## Appendix A

**Table A1:** Measuring items of constructs.

Constructs	Measuring Items
Identification with Product (IP)	IP1: Wearing Hanfu is one way to express myself.
	IP2: Hanfu fits with my style.
	IP3: I can identify with Hanfu.
	IP4: Hanfu fit with my life.
Cultural Motivation (CM)	CM1: I can increase my historical knowledge by wearing or purchasing Hanfu
	CM2: Wearing Hanfu is my way to show my interest in history.
	CM3: I can have a good time with my friends who also like Hanfu.
	CM4: I felt confident when I wear Hanfu.
	CM5: I can feel connected with the past of my country by wearing Hanfu.
Existential Authenticity (EA)	EA1: I felt connected with human history and civilization when I wear Hanfu.
	EA2: Wearing Hanfu enriched me as a person.
	EA3: By wearing Hanfu, I felt the related history and culture.
	EA4: Wearing Hanfu provided me a deeper insight of Chinese history and culture.
	EA5: I like the traditional design and cultural elements of Hanfu.
Object-based Authenticity (OA)	OA1: I like the historical meaning connected to the Hanfu.
	OA2: I like the peculiarities of Hanfu.
	OA3: The Hanfu I wear is truly historical and cultural clothing.
	OA4: The Hanfu I wear was correctly designed and produced.
	OA5: The overall Hanfu inspired me.
Purchase Intention (PI)	PI1: I will (further) purchase Hanfu in the future.
	PI2: I am planning to purchase Hanfu more frequently.
	PI3: The probability I would consider buying Hanfu is high
	PI4: I will continue purchasing and wearing Hanfu.
	PI5: I am planning to purchase Hanfu once I have a chance.

## Appendix B

**Table A2:** Results of outer loadings and cross-loadings of items.

	Identification with Product	Cultural Motivation	Object-Based Authenticity	Existential Authenticity	Purchase Intention
CM1	0.255	0.788	0.402	0.390	0.460
CM2	0.350	0.774	0.419	0.413	0.446
CM3	0.212	0.634	0.382	0.411	0.392
CM4	0.213	0.655	0.522	0.410	0.447
CM5	0.349	0.826	0.478	0.450	0.499
EA1	0.274	0.409	0.332	0.723	0.421
EA2	0.362	0.405	0.369	0.661	0.372
EA3	0.244	0.413	0.319	0.800	0.467
EA4	0.249	0.368	0.372	0.648	0.415
EA5	0.292	0.453	0.384	0.798	0.437
IP1	0.815	0.299	0.205	0.318	0.298
IP2	0.829	0.317	0.213	0.324	0.326
IP4	0.737	0.281	0.353	0.290	0.382
OA1	0.279	0.453	0.817	0.340	0.485
OA2	0.223	0.416	0.687	0.337	0.391
OA3	0.215	0.436	0.706	0.378	0.539
OA4	0.286	0.492	0.776	0.403	0.593
OA5	0.251	0.469	0.787	0.382	0.474
PI1	0.286	0.457	0.415	0.352	0.786
PI2	0.358	0.412	0.403	0.358	0.729
PI3	0.268	0.399	0.490	0.499	0.642
PI4	0.251	0.455	0.575	0.470	0.624
PI5	0.362	0.440	0.445	0.360	0.777
